# Mesenchymal stem cells maintain their defining stem cell characteristics after treatment with cisplatin

**DOI:** 10.1038/srep20035

**Published:** 2016-01-25

**Authors:** Nils H. Nicolay, Ramon Lopez Perez, Alexander Rühle, Thuy Trinh, Sonevisay Sisombath, Klaus-Josef Weber, Anthony D. Ho, Jürgen Debus, Rainer Saffrich, Peter E. Huber

**Affiliations:** 1 Department of Radiation Oncology, Heidelberg University Hospital, Im Neuenheimer Feld 400, 69120 Heidelberg, Germany; 2 Heidelberg Institute for Radiation Oncology (HIRO), National Center for Radiation Research in Oncology, Im Neuenheimer Feld 280, 69120 Heidelberg, Germany; 3 Department of Molecular and Radiation Oncology, German Cancer Research Center (dkfz), Im Neuenheimer Feld 280, 69120 Heidelberg, Germany; 4 Department of Hematology and Oncology, Heidelberg University Hospital, Im Neuenheimer Feld 410, 69120 Heidelberg, Germany

## Abstract

Mesenchymal stem cells (MSCs) aid the regeneration of tissues damaged by treatment with cisplatin. However, the effects of this cytotoxic drug on the stem cells have been largely unknown. Here we demonstrate that human bone marrow-derived MSCs are relatively resistant to cisplatin treatment and show resistance levels comparable to these of differentiated fibroblasts. Cisplatin did not affect cellular morphology, adhesion or induction of apoptosis in MSCs. The potential for differentiation was preserved after exposure to cisplatin, and established MSC surface markers were observed to be stably expressed irrespective of cisplatin treatment. Cytoskeletal rearrangements and high expression levels of individual heat shock proteins were detected in MSCs and may be partly responsible for the observed cisplatin resistance. The cisplatin-resistant phenotype of human MSCs supports the concept of further investigating these stem cells as a potential treatment option for cisplatin-induced tissue damage.

Platinum-based anticancer drugs are among the most widely used chemotherapeutic agents for the treatment of patients with solid malignancies. The first compound discovered within this group was cisplatin; it was approved for clinical use by the United States Food and Drug Administration in 1978^1^. Cisplatin has been successfully introduced into routine treatment protocols for various types of cancer, including head and neck, lung, breast, bladder, testicular, epithelial ovarian cancers, lymphomas and sarcomas^2^,^3^,^4^. However, the exact mechanism by which cisplatin exerts its effects is still incompletely understood. The drug’s cis-diammine carrier ligand has been shown to bind to DNA strands, thereby causing intrastrand and interstrand crosslinks and hence hampering DNA replication and transcription^5^. In addition to the DNA-related cytotoxic effects, cisplatin has been demonstrated to interact with other cellular structures, especially RNA molecules, membrane phospholipids and intracellular proteins^6^,^7^; it has been suggested that these interactions may also contribute to the anti-tumor effects exerted by cisplatin^8^. Cisplatin has an unfavorable toxicity profile with frequent toxicities affecting the nervous system, the kidneys and the inner ear; side effects also comprise gastrointestinal toxicity, myelosuppression and electrolyte disturbances^9^. The cisplatin-induced damage to the kidneys is commonly irreversible and usually constitutes the dose-limiting toxicity^10^.

Mesenchymal stem cells (MSCs) form a heterogeneous group of adult multipotent stromal cells that can be found in various tissues, including bone marrow, vascular and adipose tissues, skin, kidney, placenta and umbilical cord^11^,^12^,^13^. MSCs are characterized by a combination of molecular and functional features, such as their fibroblast-like appearance, their ability to adhere to plastic surfaces, their differentiation capabilities along the adipogenic, chondrogenic and osteogenic lineages and their expression of various surface markers^14^,^15^. However, no generally accepted set of MSC surface markers has been established yet, impeding the possibility to prospectively identify these cells^16^.

MSC-based treatments have been discussed as a means of repairing tissue damage, both by differentiating into organ-specific functional cells and providing a protective microenvironment^17^,^18^,^19^. Preclinical studies have widely shown a regenerative potential of MSCs, and these functions have been linked to the repair of myocardial damage, cartilage and bone injuries, pulmonary lesions as well as skin and nerve tissue damage^20^,^21^,^22^. In recent years, a potential benefit of MSCs for the repair of cisplatin-mediated tissue damage has been discussed, and animal studies demonstrated improved renal functions after MSC infusions in animal models of cisplatin-induced kidney failure^23^,^24^,^25^,^26^,^27^. However, the influence of cisplatin on the stem cells themselves remains largely unknown.

In this study, we investigated the effects of cisplatin treatment on the survival, proliferation and functional characteristics of multipotent MSCs in comparison to differentiated fibroblasts. Additionally, the influence of cisplatin on the defining stem cell properties and surface marker expression of MSCs was examined.

## Results

### MSCs and adult fibroblasts exhibit similar sensitivities to cisplatin

Cisplatin sensitivity of human MSCs and adult fibroblast cell lines HS68 and MRC5 were assessed by viability and clonogenic survival assays; the treatment doses and exposure times used in our experiments were chosen to mimic the conditions of patients undergoing cisplatin chemotherapy^28^.

**Figure 1:**
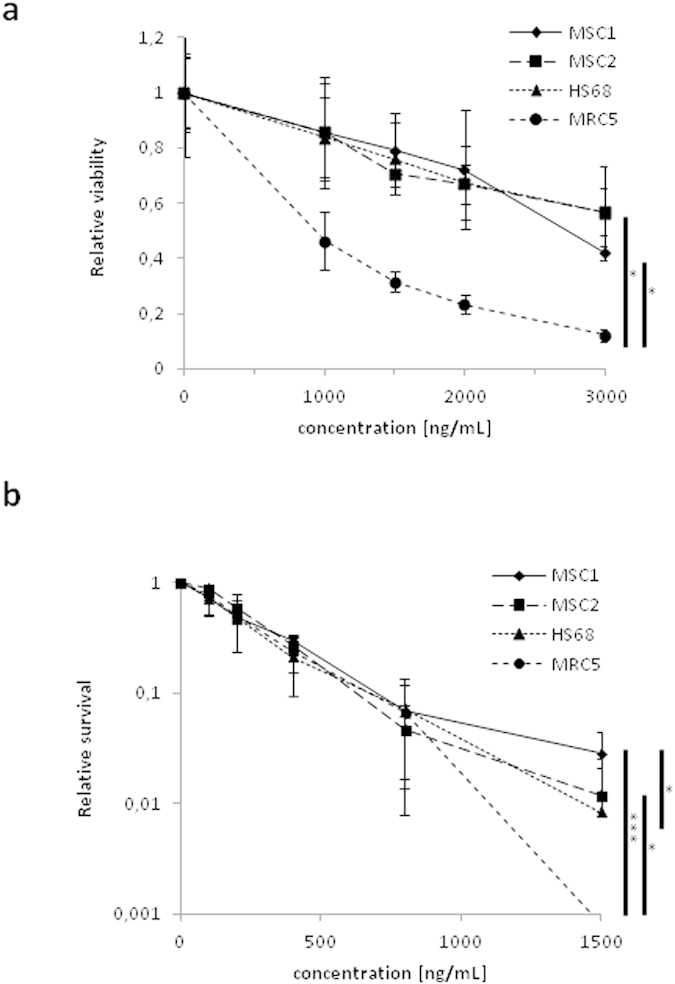
Viability and survival of mesenchymal stem cells after cisplatin treatment is comparable to differentiated fibroblasts. (**a**) MTS assay data demonstrating viability for two different MSCs and two differentiated fibroblasts after treatment with cisplatin. (**b**) Clonogenic survival assays for MSCs and fibroblasts after cisplatin treatment. Error bars represent standard deviation (n = 3). *P < 0.05, ***P < 0.001 (Student’s t-test).

After treatment with different concentrations of cisplatin, human MSCs showed no significant differences in viability compared to the cisplatin-resistant HS68 fibroblast cell line (*P* = 0.80 for MSC1, *P* = 0.59 for MSC2, two-sided Student’s t-test) and were considerably more viable than MRC5 fibroblasts (*P* < 0.05 for both MSC samples) (Fig. 1A).

Similarly, clonogenic survival data revealed that MSC1 and MSC2 samples were significantly more resistant to treatment with up to 1500 ng/mL cisplatin than MRC5 fibroblasts (*P* < 0.001 for MSC1, *P* < 0.05 for MSC2), and the MSC1 cells showed even an increased colony formation ability compared to the HS68 cell line (*P* < 0.05) (Fig. 1B).

### MSCs show no increased apoptosis after cisplatin treatment

**Figure 2:**
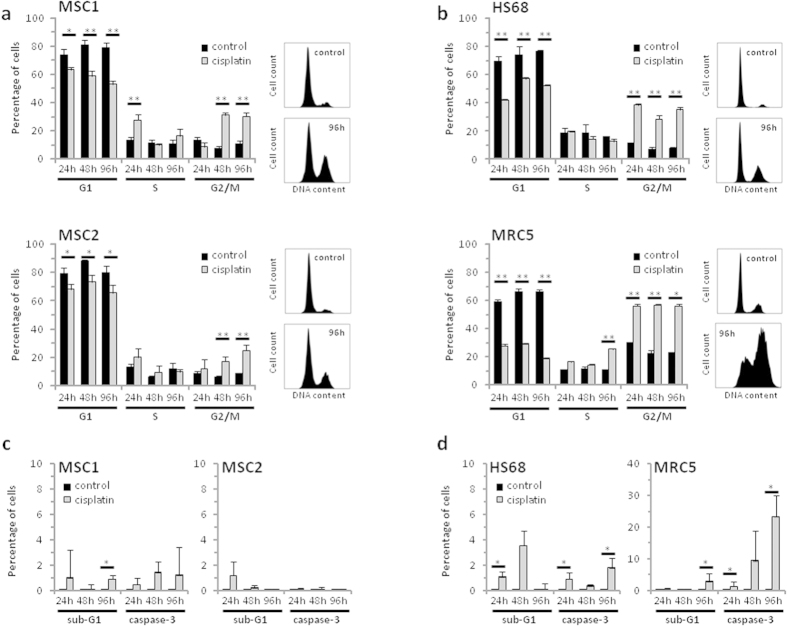
Cisplatin treatment of MSCs results in G2 phase arrest but no increase in apoptosis. Cell cycle distribution of two MSC samples (**a**) and two fibroblast cell lines (**b**) after 4-hour treatment with 1000 ng/mL cisplatin. (**c**,**d**) Percentage of apoptotic MSC1, MSC2 stem cells and HS68 and MRC5 fibroblasts after 1000 ng/mL cisplatin as assessed by sub-G1 population and caspase-3 activation. Error bars represent standard deviation (n = 3). *P < 0.05, **P < 0.01.

FACS analyses were performed to assess the influence of cisplatin treatment on human MSCs and fibroblasts. Treatment for 4 hours resulted in a prolonged G2 phase accumulation of both stem cells and fibroblasts that was present up to 96 hours (Fig. 2A,B). The observed accumulation in G2 phase occurred earlier and was more pronounced for HS68 and MRC5 fibroblasts compared to the MSC1 and MSC2 samples, correlating with their faster doubling time.

Cisplatin-induced apoptosis was assessed by measurements of cellular sub-G1 population and caspase-3 activation. Overall, MSC samples did not show a significant increase in the percentage of apoptotic cells with levels remaining below 1.5% for all tested time points (Fig. 2C). While cisplatin-resistant HS68 fibroblasts showed only a small increase in apoptosis, MRC5 cells demonstrated a strong increase in the levels of caspase-3-positive cells at later time points with 23.4% of cells in apoptosis at 96 hours after cisplatin treatment (Fig. 2D).

### Cisplatin does not impede adherence of MSCs

**Figure 3:**
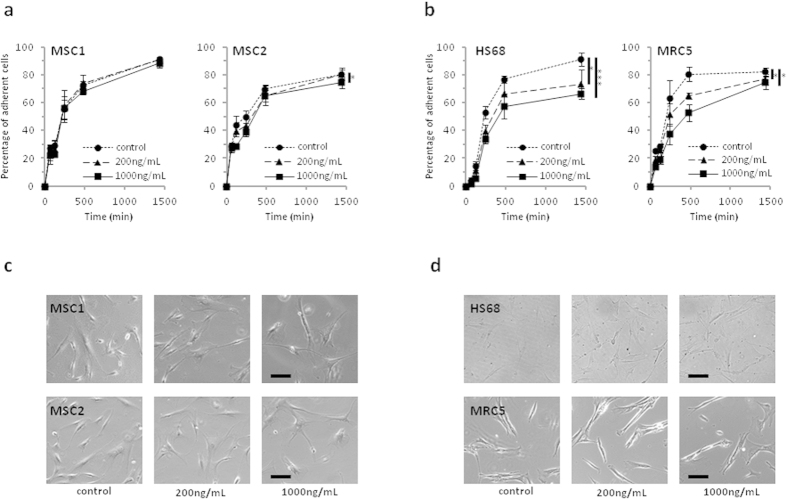
Cisplatin treatment does not impair the adhesion potential and cellular morphology of MSCs. (**a**,**b**) Relative adhesion rates of MSCs and differentiated fibroblasts up to 24 hours after 4-hour treatment with 200 or 1000 ng/mL cisplatin (n = 5). *P < 0.05, **P < 0.001 (**c**,**d**) Images of unstained MSCs and fibroblasts demonstrating no measurable changes in morphology after exposure to different cisplatin concentrations (20× objective, scale bar 100 μm).

The ability of MSCs to adhere to plastic surfaces is a defining hallmark of these stem cells; adhesion was measured up to 24 hours after treatment with cisplatin for 4 hours. Overall, adherence of MSCs was only minimally influenced by cisplatin treatment, and there was no measurable delay in adherence after treatment. At 24 hours, attachment levels of MSC1 cells treated with 200 or 1000 ng/mL cisplatin were found comparable to untreated control samples (91.0% vs. 90.8% vs. 88.4%, n.s.), while MSC2 cells showed a small but significant reduction in adherence only after treatment with high doses of 1000 ng/mL compared to the respective controls (74.6 % vs. 80.4 %, *P* < 0.05) (Fig. 3A). In contrast, HS68 cells demonstrated a dose-dependent reduction in adherence at 24 hours for treatment with 200 and 1000 ng/mL cisplatin (91.0% vs. 73.3% vs. 66.3%, *P* < 0.05 for 200ng/mL, *P* < 0.001 for 1000 ng/mL) (Fig. 3B). MRC5 fibroblasts showed a considerable dose-dependent delay in adherence; at 24 hours after cisplatin exposure, treated samples retained a small but significant reduction in adherence levels for both treatment doses (82.0% vs. 76.8% vs. 74.4%, *P* < 0.05 for 200 and 1000 ng/mL).

The morphology of MSCs and differentiated fibroblasts remained largely unchanged after treatment with 200 and 1000 ng/mL cisplatin, and no apparent morphological signs of increased apoptosis could be detected at 24 hours after treatment using light microscopy (Fig. 3C,D).

### Cisplatin treatment affects MSC but not fibroblast motility

**Figure 4:**
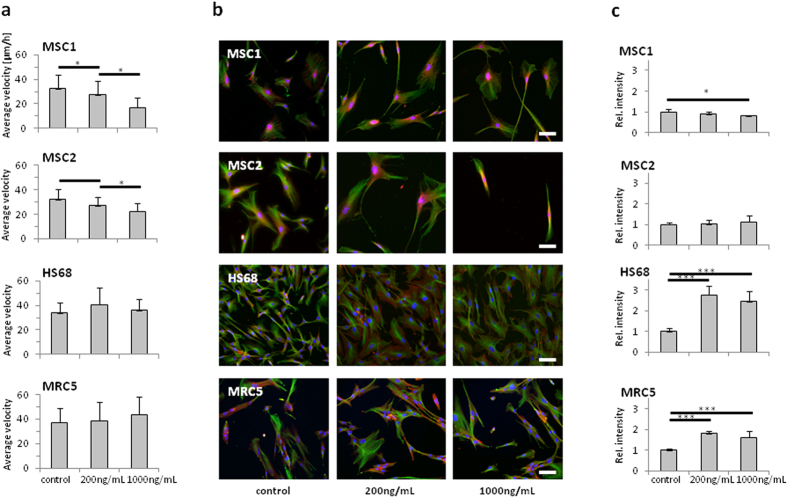
MSCs reveal reduced motility after cisplatin treatment. (**a**) Average velocity of MSCs and differentiated fibroblasts after treatment with 200 and 1000 ng/mL cisplatin. (**b**) Immunofluorescent actin and microtubule staining in MSCs and fibroblasts after cisplatin treatment (20× objective, scale bar 100 μm). (**c**) Quantification of actin staining after cisplatin treatment (n ≥ 6). *P < 0.05, ***P < 0.001.

Migration of MSCs and differentiated fibroblasts was measured by time-lapse microscopy over a time period of 24 hours. Cisplatin treatment with 200 and 1000 ng/mL for 4 hours resulted in a dose-dependent reduction of average cellular velocity for both tested MSC samples (*P* < 0.05 for 200 and 1000 ng/mL cisplatin). In contrast, the migratory ability of HS68 and MRC5 fibroblasts was not significantly altered by treatment with cisplatin (Fig. 4A).

As actin cytoskeleton dynamics have been demonstrated to correlate with the cellular motility of mesenchymally derived cells, potential alterations in the cytoskeletal architecture were assessed by immunofluorescence stainings (Fig. 4B). In MSC samples, treatment with 200 ng/mL cisplatin had no significant impact on the actin cytoskeleton. Exposure to 1000 ng/mL resulted in a small but significant reduction of the actin immunofluorescence signal in MSC1, but not MSC2 cells (*P* < 0.05 for MSC1) (Fig. 4C). In differentiated HS68 and MRC5 fibroblasts, treatment with cisplatin led to a strong increase in the actin signal independent of the treatment dose (*P* < 0.001 for 200 and 1000 ng/mL in both cell lines). No correlation could be found between the cellular motility and the detected actin cytoskeletal changes in any of the cell lines.

### Cisplatin treatment does not abolish the differentiation potential of MSCs

The ability to differentiate along the adipogenic and osteogenic lineages is a hallmark of MSCs. To investigate a potential influence of cisplatin treatment on the differentiation potential of human MSCs, immunocytochemical analyses were performed after induction of differentiation.

**Figure 5:**
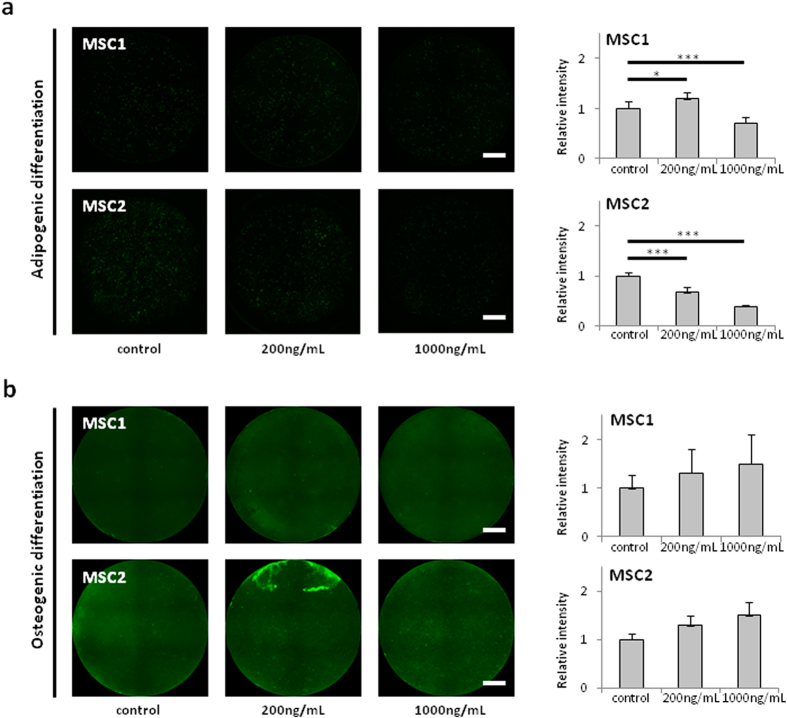
Cisplatin treatment does not abrogate the differentiation potential of MSCs. (**a**) BODIPY lipid staining in MSC1 and MSC2 samples after treatment with 200 and 1000 ng/mL cisplatin to assess adipogenic differentiation (2× objective, scale bar 2 mm). (**b**) Hydoxyapatite staining for osteogenic differentiation in MSC1 and MSC2 samples after cisplatin treatment. Relative fluorescence intensities were measured to quantify differentiation levels after adipogenic and osteogenic differentiation (n = 8). *P < 0.05, ***P < 0.001.

The adipogenic and osteogenic differentiation potential of MSC1 and MSC2 samples was found not to be abrogated even after exposure to high doses of cisplatin: While low doses of 200 ng/mL cisplatin increased the adipogenic differentiation potential of MSC1 cells (*P* < 0.05, two-sided Student’s t-test), but decreased differentiation in MSC2 samples (*P* < 0.001), higher doses of 1000 ng/mL resulted in significantly lower immunofluorescence signals for adipogenic differentiation in both MSCs (*P* < 0.001), suggesting an inhibitory effect of high cisplatin doses on MSCs’ ability to differentiate along the adipogenic lineage (Fig. 5A). In contrast, there was a trend towards dose-dependent increases in osteogenic differentiation when MSC1 and MSC2 cells were treated with different concentrations of cisplatin, although statistical significance was not reached (*P* = 0.24 for MSC1, *P* = 0.07 for MSC2 at 1000 ng/mL cisplatin) (Fig. 5B).

### Treatment with cisplatin does not affect MSC surface marker expression

**Figure 6:**
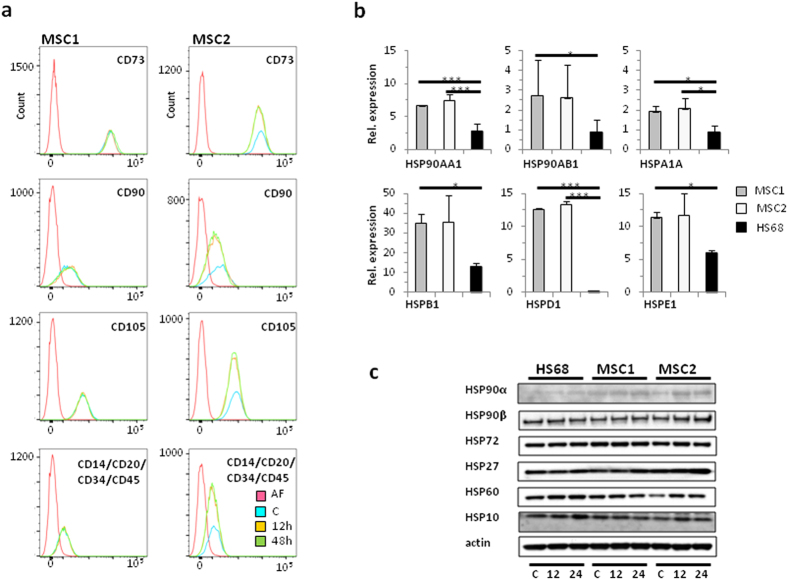
MSCs stably express defining surface markers and high mRNA levels of heat shock proteins. (**a**) Flow cytometry analyses of defining positive MSC markers CD73, CD90 and CD105 and negative markers CD14, CD20, CD34 and CD45 after treatment with 1000 ng/mL cisplatin. AF: autofluorescence, C: control, 12: 12 hours after cisplatin treatment 24: 24 hours after cisplatin treatment (**b**) Relative mRNA expression levels of different HSP genes involved in mediating cisplatin resistance. *P < 0.05, ***P < 0.001. (**c**) Western blot analyses demonstrating HSP protein expression in MSCs and HS68 fibroblasts without cisplatin treatment (**c**) and at 12 and 24 hours after treatment with 1000 ng/mL cisplatin.

Established MSC surface markers were analyzed by FACS analysis at different time points after treatment with 1000 ng/mL cisplatin. The expression patterns of positive stem cell markers CD73, CD90 and CD105 in MSC1 and MSC2 samples were not altered or reduced at 12 and 48 hours after exposure to high doses of cisplatin. Similarly, cisplatin treatment had no influence on the expression of the hematopoietic negative markers, CD14, CD20, CD34 and CD45 (Fig. 6A).

### MSCs exhibit high expression levels of heat shock proteins

High expression levels of several heat shock proteins (HSPs) have been linked to increased resistance to cisplatin. Gene array data obtained from MSC1 and MSC2 samples and HS68 fibroblasts revealed high mRNA levels of HSP90AA1 and HSP90AB1, encoding for the two cytosolic HSP-90 proteins α and β (Fig. 6B). Similarly, MSCs exhibited high expression of HSP genes, HSPA1A, HSPB1, HSPD1 and HSPE1, encoding for HSP-72, HSP-27, HSP-60 and HSP-10, respectively. Levels of HSP90AA1, HSPA1A and HSPD1 were found to be significantly higher in both MSC specimens, while HSP90AB1, HSPB1 and HSPE1 were significantly increased only in MSC1 cells compared to HS68 fibroblasts. Compared to the transcriptional level, HSP expression appeared considerably more equal between MSCs and the fibroblast line on the protein level; Western blot data demonstrated notable differences between stem cells and fibroblasts only for HSP90α and HSP27. Moreover, cisplatin treatment resulted in an upregulation of several HSPs in particular in MSCs. (Fig. 6C).

## Discussion

While MSCs may exert their regenerative effects on tissues damaged by cisplatin-based chemotherapy, the effect of the cytotoxic drug on the stem cells themselves has been largely unknown. The data presented here demonstrated that MSCs were relatively resistant to treatment with cisplatin and retained levels of viability and colony formation ability comparable to those of cisplatin-resistant fibroblasts^29^. The treatment doses and exposure times used in our experiments were chosen to mimic the conditions of patients undergoing cisplatin chemotherapy, where plasma cisplatin levels usually range below 1500 ng/mL with a plasma half-life below 1 hour^28^,^30^.

Earlier *in-vitro* analyses provided inconsistent data regarding the sensitivity of MSCs against different anticancer agents including camptothecin, vincristine, ionizing radiation and targeted kinase inhibitors^31^,^32^,^33^,^34^. However, bone marrow samples harvested from cancer patients treated with cisplatin, vincristine or etoposide were shown to contain viable and proliferating MSCs, suggesting a relative resistance *in vivo*^35^. Additionally, MSCs were found to be relatively resistant to cisplatin compared to hematopoietic stem cells and leukemia cell lines^36^. In this context of an observed cisplatin-resistant phenotype, our analyses demonstrated that MSCs did not undergo apoptosis even after treatment with high doses of cisplatin. This resistance against apoptotic activation has been reported after other forms of cytotoxic treatment^37^, and it has been suggested that the observed resistance of MSCs is due to a reduced p73-dependent activation of pro-apoptotic proteins, p21 and Bax and high constitutive expression levels of various anti-apoptotic proteins such as Bcl-2 and Bcl-xL^38^,^39^. Additionally, MSCs were shown to lack activation of the TRAIL pro-apoptotic pathway upon cisplatin treatment^40^.

While cisplatin resistance has been reported to be associated with increased motility in different tumor cell lines^41^, we observed a dose-dependent decrease in velocity of MSCs after exposure to high doses of cisplatin that was not present in differentiated fibroblasts. The organization of the actin cytoskeleton has been linked to the ability of mesenchymal cells for locomotion^42^. Our immunocytochemical analyses of the filament system revealed a small reduction in the cytosolic actin levels in MSC1, but not MSC2 samples. In contrast, the assessed fibroblasts showed a strong, dose-independent increase in actin after exposure to cisplatin. The influence of cisplatin on the expression and structure of actin microfilaments has previously been demonstrated: While cisplatin treatment was described to result in the collapse of the actin filament system in different cell types, cisplatin-resistant cells were found to exhibit reduced actin levels^43^,^44^. In addition, super-resolution microscopy demonstrated radial re-arrangements of actin microfilaments around the nucleus in cisplatin-resistant ovarian cancer cells, correlating with an increased stiffness of the cells^45^. Similar perinuclear staining patterns for actin were detected in our MSC samples. In contrast, both HS68 and MRC5 fibroblasts treated with cisplatin exhibited an increased distribution of the actin immunofluorescence signal throughout the cytosol, correlating with an overall increase in actin detection. It has been shown previously that actin remodeling and increased actin signals were associated with cisplatin-induced apoptosis^46^, and indeed we detected a higher percentage of both tested fibroblast cell lines undergoing apoptosis after cisplatin treatment compared to MSCs. The observed reduction of MSC velocity after treatment with cisplatin may be of therapeutic relevance: MSCs have been shown to move towards and integrate into tissues damaged by cancer treatments, where they aid organ repair^47^,^48^. This ability to home into tissue lesions may be impaired after cisplatin-based therapies, and cisplatin damage may be augmented by the inability of endogenous MSCs to participate in the organ regeneration. In line with this hypothesis, infusions of exogenous cisplatin-naïve MSCs may substitute the impaired function of endogenous stem cells, and animal studies have demonstrated increased repair of cisplatin-induced lesions in different organs, including kidney and ovaries^27^,^49^,^50^.

Beyond their relative resistance against cisplatin treatment, we demonstrated that MSCs maintained their defining stem cell properties and surface marker profiles even after exposure to high doses up to 1000 ng/ml of cisplatin. The ability to adhere to plastic surfaces is a key feature of MSCs and is often used to select these stem cells in culture. MSC adherence was only marginally altered after high-dose cisplatin treatment, and we observed no delay in adherence compared to untreated control samples. The adhesion potential of MSCs has previously been demonstrated to be unaffected by other forms of DNA damaging agents, and these findings were corroborated by the upregulation of various genes involved in cellular adhesion of MSCs^32^,^51^.

Likewise, the ability to undergo induced differentiation along the adipogenic and osteogenic lineages constitutes a defining trait of MSCs; however the influence of cisplatin on this differentiation potential has remained unknown. Here, we demonstrated for the first time that the differentiation potential of MSCs was preserved even after exposure to high doses of cisplatin, and we did not find a general dose-dependent reduction of the differentiation capabilities: While the adipogenic differentiation was found to be reduced at least after treatment with high doses of cisplatin, MSCs showed a trend towards increased osteogenic differentiation after cisplatin treatment. This finding may be of special therapeutic relevance, as several publications have provided evidence for the regenerative effects of autologous MSC treatments after cisplatin-induced tissue damage^23^,^24^. Therapies using exogenous MSCs to treat cisplatin-induced tissue damage may not be directly dependent on the described cisplatin resistance of these cells, as MSCs are usually only detectable for few days; however, accumulation of cisplatin has been demonstrated in human tissue autopsy specimens up to several months after treatment and may potentially still affect exogenous, cisplatin-naïve MSCs^52^,^53^. Additionally, the mobilization of endogenous MSCs may play an important physiological role in the repair of cisplatin-induced tissue damage. It has been suggested that MSCs can differentiate into different types of functional cells within damaged target tissues, further aiding the regeneration of organ lesions caused by cisplatin^54^. This repair capacity is directly dependent on the ability of these cells to survive and maintain their regenerative properties^54^. Therapeutic approaches utilizing a mobilization of endogeneous MSCs to treat cisplatin-induced tissue damage are yet to be tested *in vivo*.

Several heat shock proteins (HSPs) have been linked with increased cisplatin resistance both *in vitro* and *in vivo*. Overexpression of HSP27, HSP60, HSP70 and HSP90 was shown to protect both tumor and renal tissues against cisplatin^55^,^56^,^57^,^58^, and high levels of HSP10 and HSP70 were found to reduce levels of cisplatin-induced apoptosis^59^,^60^. We observed high constitutive mRNA expression levels for HSP90AA1 and HSP90AB1, HSPA1A, HSPB1, HSPD1 and HSPE1, encoding for HSP-90 α and β, HSP-72, HSP-27, HSP-60 and HSP-10 proteins, respectively. Several publications have shown a sensitization of cells and tissues to cisplatin upon HSP inhibition, further strengthening the link between HSP expression and cisplatin resistance^61^,^62^. In fact we found that cisplatin treatment resulted in increased levels of several HSPs in particular in MSCs, suggesting that HSPs may be involved in MSC resistance to cisplatin. However, HSP protein levels appeared considerably more equal between MSCs and fibroblasts, and only HSP90α and HSP27 protein expression appeared higher in the tested stem cells compared to the fibroblast cell line. Additionally, HSP protein levels were found to increase upon cisplatin treatment in the MSC samples. Therefore, it is conceivable that the observed relative resistance of MSCs against cisplatin treatment in our dataset may at least in part be due to the detected high levels of protective HSPs.

Taken together, our findings characterized the cisplatin-resistant phenotype of human MSCs and demonstrated that these stem cells maintained their defining cellular properties even after treatment with high doses of cisplatin. Highly expressed HSPs may at least partly mediate this observed cisplatin resistance.

## Materials and Methods

### Cells and cultures

Primary human MSC1 and MSC2 mesenchymal stem cells were harvested from bone marrow samples of healthy donors and isolated as previously published^37^,^63^. MSCs were proliferated in Mesenchymal Stem Cell Growth Medium (*MSCGM*^*TM*^, Lonza, Basel, Switzerland), supplemented with *MSCGM*^*TM*^ SingleQuots (Lonza) and were kept in a humidified incubator at 37 °C and 5% CO_2_. MRC5 human pulmonary fibroblasts were purchased from the ATCC (Manassas, USA) and were maintained in Eagle’s Minimum Essential Medium supplemented with 10% fetal bovine serum. Human HS68 dermal fibroblasts were obtained from the ATCC and proliferated in Dulbecco’s Modified Eagle Medium (Biochrom, Berlin, Germany); 10% fetal bovine serum and 3,5 g/L glucose were added to the medium. For the MSCs, written consent from donors was obtained before the harvesting according to current ethics guidelines. This study was approved by the independent ethics board of the University of Heidelberg, and all experiments were carried out in accordance with the approved guidelines.

### Drug preparation

Cisplatin stock solution was obtained from the Heidelberg University Hospital central pharmacy and was stored in the refrigerator for up to 7 days. Immediately prior to each experiment, the drug was diluted in culturing medium to the required concentrations. All experimental setups containing cisplatin were protected from light.

### Viability assays

The MTS assay was used to assess cellular viability after drug treatment. 2000 cells were plated in each well of a 96-well plate containing 200 μL of medium, and cisplatin was added to the wells at concentrations ranging between 1000 and 3000 ng/mL; cells were then incubated for 5 days. 20 μL of 1.9 mg/mL MTS reagent (Promega, Madison, USA) was added to each well and further incubated for 2 hours before absorbance at 490 nm was measured on a microplate reader (Tecan, Crailsheim, Germany).

### Clonogenic survival assays

Cells were plated and left to attach for 6 hours before treatment. Cisplatin concentrations between 100 and 1500 ng/mL were added to the cells for 4 hours before the replacement of medium. After drug treatment, cells were maintained for 14 days to enable colony formation. Colonies were then fixed with 25% acetic acid (v/v) in methanol and stained with crystal violet solution. Colonies containing in excess of 50 cells were counted using a light microscope. All clonogenic assays were performed in triplicate. The surviving fraction of cells was calculated by the following formula: (#colonies/#plated cells)_treated_/(#colonies/#plated cells)_untreated_.

### Cell cycle analyses

To investigate cell cycle profiles, cells were harvested after a 4-hour treatment with 1000 ng/mL cisplatin and washed before fixation in ice-cold 70% ethanol. After centrifugation, cells were incubated with 1 μg/mL 4′,6-diamidin-2-phenylindol (DAPI) solution containing 200 μg/mL RNase A. Flow cytometry analysis was then performed on a LSR II system (Becton-Dickinson, Heidelberg, Germany). 10000 events were counted for each experimental condition, and cell cycle profiles were modeled using FlowJo 7.6.5 software (FlowJo LLC, Ashland, USA).

### Apoptosis measurements

Cells were harvested after treatment with 200 and 1000 ng/mL cisplatin for 4 hours and fixed in 4% paraformaldehyde solution before resuspension in ice-cold 70% ethanol. Cells were then washed in PBS containing 200 μg/mL RNase A and 5 g/L bovine serum albumin. After centrifugation, cells were incubated with a fluorescence-coupled antibody against activated caspase-3 (1:20, BD Pharmingen, Heidelberg, Germany) for 1 hour at room temperature. Flow cytometry analyses were then carried out using a LSR II system. 10000 events were counted for each experimental condition.

### Adhesion measurements

Cells were treated with cisplatin for 4 hours at the concentrations indicated in the Results section. 100 cells were then plated in each well of 96-well plates, and attached cells were counted on a light microscope at different time points after plating. The attachment efficiency was calculated as the ratio between attached and plated cells. All measurements were performed at least in triplicate.

### Migration measurements

Cells were grown in 24-well plates to a confluence of 30–50% and treated with 200 or 1000 ng/mL cisplatin for 4 hours before migratory behavior of MSCs and differentiated fibroblasts was measured every 5 minutes over a time period of 24 hours by time-lapse microscopy. Imaging was performed on an IX70 inverted microscope equipped with an incubator box (Olympus, Hamburg, Germany). Quantification of cellular migration was assessed by manual single-cell tracking using ImageJ software (National Institutes of Health, Bethesda, USA). For each experimental condition, tracks of at least 10 cells from three locations in each well were measured.

### Immunocytochemistry

The MSC cytoskeletal architecture was analyzed by immunocytochemistry. After fixation with 4% PFA, cells were permeabilized with 0.2% Triton X-100 for 5 min and blocked with 5% NGS for 1 hour to reduce unspecific binding of antibodies. Actin filaments were visualized by incubating the cells with 100 nM of Alexa Fluor® 633 phalloidin (Life Technologies, Darmstadt, Germany) in PBS for 30 min. For microtubule staining, cells were incubated with a primary mouse monoclonal anti-a-tubulin antibody (SIGMA, Munich, Germany) for 1 hour, followed by three washing steps with PBS. Secondary anti-mouse antibodies conjugated with DyLight® 488 (abcam, Cambridge, UK) were then added for 1 hour. A nuclear staining was carried out using 1 μM DAPI for 5 min. For quantification, at least five images were aquired for each treatment condition with a Keyence BioRevo9000 microscope (Keyence, Neu-Isenburg, Germany) using a 20× objective. Data analysis was performed with ImageJ software (National Institutes of Health, Bethesda, USA).

### Cellular differentiation experiments

To assess the differentiation potential of MSCs, log phase cells were plated in 24-well plates and treated with 200 or 1000 ng/mL cisplatin for 4 hours. At 24 hours after treatment, medium was replaced by differentiation media, and cells were grown for 21 days. All differentiation media were exchanged twice weekly. Adipogenic differentiation media consisted of DMEM supplemented with 10% FCS, 2 mM L-glutamine, 1 μM dexamethasone, 500 μM 1-methyl-3-isobutylxanthine, 10 μg/mL insulin and 100 U/mL penicillin/streptomycin. To detect adipogenic differentiation, cells were incubated with 1 μg/ml BODIPY (493/503) (Life Technologies, Darmstadt, Germany) for 20 min. After 3 washing steps with PBS, nuclei were stained with 1 μM DAPI for 5 min.

Osteogenic differentiation was induced using DMEM containing 10% FCS, 10 mM b-glycerophosphate, 1 μM dexamethasone, and 0.2 mM ascorbic acid. Differentiated cells were stained with OsteoImage™ Staining Reagent (Lonza, Cologne, Germany) according to the manufacturer’s protocol; nuclear staining was carried out with 1 μM DAPI for 5 min. For the analysis of differentiation, fluorescence images of whole wells of the 24 well plate were obtained for all experiments under identical conditions using a Keyence BioRevo9000 microscope. The mean fluorescence intensity of each well was measured and quantified using ImageJ software.

### Surface marker measurements

MSCs were grown in parallel in 75 cm^2^ flasks and 25 cm^2^ flasks up to 80% confluency. Then cells in the 25 cm^2^ flasks were treated with cisplatin at a concentration of 1000 ng/mL for 4 h. At 12 and 48 hours after treatment, cells were harvested and examined for surface markers on the basis of the proposed minimal criteria for MSCs^15^, using the MSC Phenotyping Kit (Miltenyi Biotec, Bergisch-Gladbach, Germany).)The staining was performed according to the manufacturer’s instructions. Measurements were done on a FACSCanto flow cytometer (Becton-Dickinson, Heidelberg, Germany) followed by data analysis using FlowJo 7.6.5 software.

### Gene expression analysis

Gene expression patterns of MSCs and HS68 fibroblasts were analyzed using a whole human genome microarray 4 × 44 k (Agilent Technologies, Böblingen, Germany). RNA was extracted from log-phase cells using an RNeasy Mini Kit (Qiagen, Hilden, Germany). Data were extracted with the Agilent feature extraction software (version 9.1) and assessed. Statistical analysis was performed using the paired Student’s t-test.

### Western blot analyses

MSCs were treated with 1000 ng/mL cisplatin for 4 hours, and cells were harvested 12 and 24 hours later. Each sample containing 10 μg of total protein from whole-cell lysates was run on a polyacrylamide gel and transferred to a polyvinylidene difluoride membrane (Millipore, Darmstadt, Germany). Membranes were probed with primary antibodies against HSP90α (Abcam, Cambridge, UK), HSP90β (Abcam), HSP72 (LifeSpan Biosciences, Eching, Germany), HSP27 (Cell Signaling Technology, Leiden, Netherlands), HSP60 (Cell Signaling Technology), and HSP10 (Santa Cruz, Heidelberg, Germany). β-actin was used as a loading control. Blots were visualized on X-ray film using a horseradish-peroxidase kit (Cell Signaling Technology).

## Additional Information

**How to cite this article**: Nicolay, N. H. *et al*. Mesenchymal stem cells maintain their defining stem cell characteristics after treatment with cisplatin. *Sci. Rep.* **6**, 20035; doi: 10.1038/srep20035 (2016).
